# Iron Deficiency Anaemia Screening and Management in Young Children: India and Southeast Asia Consensus

**DOI:** 10.1155/anem/6347066

**Published:** 2025-11-17

**Authors:** Muhammad Yazid Jalaludin, Hamid Jan Jan Mohamed, Sri Wahyu Taher, Kim Ang, Lam Pechkethia, Suchaorn Saengnipanthkul, Ketkesone Phrasisombath, Alongkone Phengsavanh, Reeta Bora, Sunil Kumar Agarwalla

**Affiliations:** ^1^ Department of Paediatrics, Faculty of Medicine, University Malaya, Lembah Pantai, Kuala Lumpur, 59100, Malaysia, um.edu.my; ^2^ Nutrition Programme, School of Health Sciences, Universiti Sains Malaysia, Kubang Kerian, 16150, Kelantan, Malaysia, usm.my; ^3^ Klinik Kesihatan Simpang Kuala, Alor Setar, 05400, Kedah, Malaysia; ^4^ National Paediatric Hospital, Phnom Penh, 120404, Cambodia; ^5^ Department of Haematology, National Paediatric Hospital, Phnom Penh, 120404, Cambodia; ^6^ Department of Paediatrics, Faculty of Medicine, Khon Kaen University, Khon Kaen, 40002, Thailand, kku.ac.th; ^7^ Department of Hygiene and Health Promotion, Ministry of Health, Vientiane, 01000, Laos, moh.gov.la; ^8^ Department of Obstetrics and Gynaecology, Faculty of Medicine, University of Health Sciences, Vientiane, Laos, uhs.edu.pk; ^9^ Department of Paediatrics, Assam Medical College, Dibrugarh, 786001, India, assammedicalcollege.in; ^10^ Department of Paediatrics, SCB Medical College and Sishubhawan Sardar Vallabhbhai Patel Post Graduate Institute of Paediatrics, Cuttack, 753001, India

**Keywords:** IDA management, IDA prevention, IDA screening, iron deficiency anaemia, paediatric anaemia

## Abstract

Iron deficiency anaemia (IDA) remains highly prevalent among children in India and many Southeast Asian countries. Experts in maternal and child health have developed a consensus on IDA in children. This consensus aims to improve awareness of IDA, provide recommendations on the screening and management of children at risk of IDA and offer insights into strategies to prevent IDA in children. The consensus was developed using the Delphi method, where eight primary expert members initially formulated questions on IDA screening and management based on a comprehensive literature review, followed by feedback and voting from 18 secondary expert members, achieving consensus with at least 70% agreement. Twelve statements achieved consensus to provide guidance and recommendations on several key areas: the recommended age for initial and annual anaemia screening, the use of noninvasive haemoglobin measurement devices for screening, further evaluations to rule out thalassaemia and IDA in children with anaemia and preventative measures to reduce the risk of IDA. The experts agreed that early detection of anaemia is crucial to mitigate related health consequences in children. It is recommended that all children undergo their first screening for anaemia between the ages 9 and 12 months, followed by annual screenings from the ages 1 to 5 years. In addition, the experts emphasised the importance of nutritional intervention, particularly the fortification of food and milk, to assist in reducing the risk of childhood IDA. By integrating relevant recommendations based on current clinical data and best practices, these consensus statements serve to guide screening, treatment and prevention of IDA among children.

## 1. Introduction

Most anaemic children are asymptomatic and can be easily missed in clinical settings [1]. Moderate‐to‐severe anaemia is known to cause breathlessness, fatigue, delayed cognitive development, impaired growth and impaired cardiac function in children [2]. According to the World Health Organization (WHO), anaemia in children is defined as < 10.5 g/dL for ages 6–23 months, < 11 g/dL for ages 24–59 months and < 11.5 g/dL for ages 5–11 years [3]. A large proportion of children aged 6–59 months in India and Southeast Asia are affected by anaemia, with the highest prevalence recorded in India (53.4%), followed by Myanmar (49.6%) and Cambodia (49%) [4].

While anaemia is often multifactorial, the WHO recognises iron deficiency as the primary cause of anaemia, attributing to approximately half of the global anaemia cases [2]. In infants, iron deficiency may arise from maternal iron deficiency, prematurity or low birth weight, exclusive breastfeeding beyond 6 months and delayed or insufficient introduction of iron‐rich solids [5]. Meanwhile, iron deficiency in children may stem from dietary practices such as vegetarianism or a vegan diet, or high consumption of foods rich in phytates or polyphenols [6]. Other causes for anaemia in children include micronutrient deficiencies [2], helminth infection [7] and thalassaemia [8]. In addition, socioeconomic determinants including poverty, food insecurity, poor sanitation and limited awareness of iron deficiency anaemia (IDA), particularly in rural, peri‐urban and slum areas, may also place children in India and Southeast Asia at heightened risk for IDA [9, 10].

Due to the impact of untreated and undiagnosed IDA on a child’s long‐term health, early diagnosis is crucial to prevent undesirable health consequences [11]. Unfortunately, anaemia screening is often overlooked due to the invasive nature of blood sampling required to test for serum haemoglobin, haematocrit or ferritin levels. Furthermore, although diagnosing IDA through a bone marrow biopsy is considered the gold standard, this invasive procedure is typically not performed until a child exhibits severe symptoms [10]. These factors combined have led to an unsatisfactory screening rate for IDA.

This expert consensus on IDA screening and management in children was developed to address limited awareness and underrecognition of IDA, underdiagnosis of IDA in children due to invasive screening methods and suboptimal implementation of prevention strategies in the region. Experts in maternal and child health from India and Southeast Asia have established a consensus statement outlining insights on IDA. The objectives of this expert consensus are to improve awareness of IDA in children, to develop specific recommendations on screening and management of children at risk of IDA and to support the prevention of IDA in children through screening, nutrition management and caregiver education.

## 2. Methodology

This consensus was developed according to the Delphi method, featuring an iteration and controlled feedback process that continued until consensus was reached for all statements [12]. The primary expert panel consisted of eight healthcare providers, including three paediatricians, two nutritionists, a family medicine specialist, a public health specialist and an obstetrician and gynaecologist. This international group of experts were gathered from India and four Southeast Asian countries: Malaysia, Cambodia, Thailand and Laos. Invitations were extended to experts from the Philippines and Indonesia, but no responses were received from experts in the Philippines, while experts in Indonesia had declined to participate as a similar initiative already existed within the country.

The primary research question was developed based on the authors’ interest in screening and managing IDA in young children within the region, guided by the population, intervention, professionals, outcome and healthcare setting (PIPOH) framework [13]: What are the strategies to strengthen IDA screening and management in young children at the community level in India and SEA? Following that, five secondary research questions on the screening and management of IDA in children aged 1–5 years were identified:

• Question 1: Why is early detection of anaemia in children important?
• Question 2: At what age should children be screened for anaemia?
• Question 3: How often should children be screened for anaemia?
• Question 4: How to screen children for anaemia?
• Question 5: What strategies can be implemented to prevent and manage IDA from pregnancy through childhood? What strategies and government policies are in place to improve iron intake in children?

A literature review of existing evidence on screening and management of IDA in children was conducted on PubMed and Google Scholar. Keywords used in the search strategy included terms related to the population (child, infant and paediatric), condition (anaemia, iron deficiency and IDA) and key concepts (screening, early diagnosis, early detection, checkup, prevention, preventive strategies, iron supplement, iron fortification, management and treatment). Inclusion criteria comprised literature relevant to India and Southeast Asia, published from the year 2000 up to May 2023, when the search was last performed. Eligible articles focused on children with IDA and included randomised controlled trials, systematic reviews, meta‐analyses and observational studies, with abstracts in English. Additional relevant literature was identified from the reference lists of included articles, as well as from grey literature and clinical guidelines in English and their local language.

Ten consensus statements addressing the research questions were then developed based on available literature and informed by the authors’ clinical and research expertise. The consensus statements were deliberated by the primary expert members during an in‐person meeting held in Bangkok, Thailand. An anonymous live voting poll using SurveyMonkey was held during the meeting, where panel members indicated their level of agreement for each statement: ‘Agree’, ‘Maybe’ or ‘Disagree’. Members who voted ‘Maybe’ or ‘Disagree’ were encouraged to disclose their reasons for uncertainty or disagreement for further discussions. Revisions were made as agreed upon by the primary expert members, with two additional statements formulated through expert discussion and supported by findings from the literature review. Subsequently, the primary expert members convened for a virtual meeting to finalise their level of agreement for 12 agreed‐upon statements.

**Figure 1:**
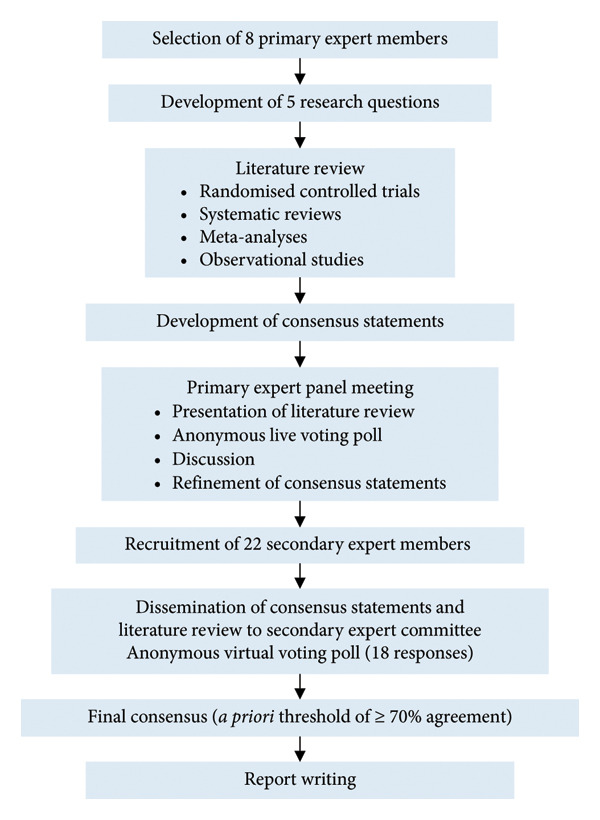
Consensus process for the development of this expert consensus on iron deficiency anaemia screening and management in young children.

The primary expert members nominated 22 healthcare providers with specialisation in paediatrics, nutrition and family medicine from India and all Southeast Asian countries (Malaysia, Thailand, Laos, Myanmar, Cambodia and the Philippines) to participate in the second round of consensus statement voting. The consensus statements and literature review were disseminated to the secondary group, together with an anonymous virtual voting poll. The secondary expert members selected their level of agreement with each statement and commented on their reasons should they not fully agree with a particular statement. A total of 18 experts completed the second voting poll. Consensus was signified by an a priori agreement level of at least 70% [14]. The flow of consensus development via the Delphi technique is shown in Figure 1.

## 3. Results

The consensus statements for optimising the screening and management of IDA in children from India and Southeast Asia, along with the agreement ratings at the first and second rounds of voting, are presented in Table 1. Most of the consensus recommendations achieved unanimous agreement (100%) between the primary and secondary expert members. Statements that did not achieve 100% agreement still exceeded the a priori of 70% agreement.

**Table 1:** Consensus statements and their respective agreement ratings from two rounds of voting by the primary and secondary expert groups.

Consensus statements	Agreement (%)		Mean agreement (%)
	Primary expert group	Secondary expert group	
Consensus statement 1: Anaemia should be detected early to reduce related health consequences in children.	100	100	100
Consensus statement 2: All children are recommended to undergo first screening for anaemia between 9 and 12 months of age.	100	83.3	91.7
Consensus statement 3: All children aged 1–5 years should be screened for anaemia annually.	100	88.9	94.5
Consensus statement 4: Screening for anaemia should be done using a noninvasive haemoglobin measurement device.	87.5	77.8	82.7
Consensus statement 5: Children who are detected to be anaemic using noninvasive screening should have further evaluation.	100	100	100
Consensus statement 6: As thalassaemia is prevalent in India and Southeast Asia, further test(s) should be performed accordingly in children found to have anaemia.	100	94.4	97.2
Consensus statement 7: Iron deficiency anaemia is prevalent in India and Southeast Asia; hence, iron deficiency should be ruled out in children found to have anaemia.	100	100	100
Consensus statement 8: Nutritional intervention, particularly food fortification, should be introduced to all children to reduce the risk of IDA.	100	83.3	91.7
Consensus statement 9: Active caregiver education on IDA, its risks and ways to reduce the likelihood of developing IDA is recommended.	100	100	100
Consensus statement 10: Improving women’s iron status from prepregnancy, during pregnancy and lactation is recommended to reduce IDA risk in children.	100	94.4	97.2
Consensus statement 11: Deworming in helminth‐endemic areas is encouraged to curb iron loss among children.	100	100	100
Consensus statement 12: Children found to have IDA should receive iron treatment in addition to other measures to prevent or treat IDA.	100	94.4	97.2

## 4. Discussion

### 4.1. Question 1: Why Is Early Detection of Anaemia in Children Important?

#### 4.1.1. Consensus Statement 1: Anaemia Should Be Detected Early to Reduce Related Health Consequences in Children

In young children, IDA can adversely impact their cognitive performance, behaviour and physical growth, weaken the immune system and increase morbidity from infections, as well as limit their physical capability and school performance [15]. Children affected by IDA often develop symptoms including impaired cognitive function, memory loss, difficulties in learning and concentration, fatigue and behavioural disturbances [5]. Therefore, the experts unanimously agreed (100%) that early detection of anaemia is important to prevent its associated health consequences in children.

### 4.2. Question 2: At What Age Should Children Be Screened for Anaemia?

#### 4.2.1. Consensus Statement 2: All Children Are Recommended to Undergo First Screening for Anaemia Between 9 and 12 Months of Age

The American Academy of Pediatrics (AAP) advocates universal screening with haemoglobin determination for anaemia at age 1 year [16]. Universal screening should also include a comprehensive assessment of risk factors associated with iron deficiency or IDA such as evaluating history of prematurity or low birth weight, lead exposure, prolonged exclusive breastfeeding beyond 4 months without supplemental iron or transition to whole milk or complementary foods low in iron [16]. Thorough history taking is critical in screening, as most children with mild anaemia may not present with visible symptoms [17]. If suspected to be anaemic, blood tests are recommended to diagnose IDA using laboratory parameters like serum ferritin, C‐reactive protein or serum transferrin receptor 1 [16]. Children at any age with risk factors for anaemia are also advised for selective screening, including feeding difficulties, impaired growth and insufficient dietary iron intake [16].

A consultation meeting jointly organised by the United Nations Children’s Fund (UNICEF), United Nations University (UNU) and WHO recommended assessing iron status based on resource availability within a country [15]. Countries with adequate to intermediate levels of resources are recommended to screen using haemoglobin and haematocrit tests, whereas in resource‐limited settings, clinical assessment may suffice to identify more severe anaemia cases, particularly when laboratory testing is not feasible [15]. The recommended methods for determining the prevalence of anaemia via haemoglobinometry include the cyanmethemoglobin method and the HemoCue (HemoCue, Ängelholm, Sweden) system [15]. UNICEF, UNU and WHO also jointly recommend initiating anaemia screening when a country’s anaemic prevalence exceeds 5% [15].

Several countries in Asia have established regulations for anaemia screening at the local level [18–21]. In India, children aged 6 months to 5 years undergo opportunistic screening, conducted either through clinical examination or haemoglobin tests during village health and nutrition day, immunisation sessions, house‐to‐house visits by accredited social health activists or when a sick child visits a health facility [18]. The Royal College of Pediatricians of Thailand recommends screening infants for haemoglobin or haematocrit levels at 9 months of age [21]. Meanwhile, Indonesia does not specify screening recommendations [22], relying instead on established laboratory tests (Sekartini et al.). In Cambodia, guidelines from the National Pediatric Hospital recommend using complete blood counts or serum iron and ferritin levels to screen for IDA [19]. Currently, there are no specific guidelines for anaemia or IDA screening in Malaysia, Vietnam or Laos.

This consensus recommends initial screening for anaemia in all children between 9 and 12 months because the experts agreed that, at this age, children who are exclusively breastfed or breastfed without sufficient iron supplement are at the highest risk of IDA. Initial screening can also be conducted between 6 and 9 months if it better fits the local national immunisation schedule or performed as early as 6 months old in children at risk of developing anaemia.

During the second voting, three experts did not concur (83.3% agreement, two voted ‘Maybe’ and one voted ‘Disagree’) with undergoing first screening for anaemia between 9 and 12 months because: (i) high‐risk infants may require earlier screening and (ii) iron deficiency may occur earlier for exclusively breastfed infants without supplemental iron. These concerns were addressed with AAP recommendations to conduct selective screening at any age for children with risk factors for anaemia [16].

### 4.3. Question 3: How Often Should Children Be Screened for Anaemia?

#### 4.3.1. Consensus Statement 3: All Children Aged 1–5 Years Should Be Screened for Anaemia Annually

International and national guidelines recommend periodic anaemia screening in children over 1 year old [16, 18, 21, 23, 24]. The AAP advises additional anaemia screening between ages 1 and 5 for at‐risk children, including those with feeding problems, poor growth, inadequate nutrition or low socioeconomic status [16, 23]. In China, primary healthcare facilities conduct annual screening for anaemia [24], whereas India recommends opportunistic screening in children up to 5 years old [18]. The Royal College of Pediatricians of Thailand also suggests rescreening children at ages 3–5 years following the initial screening at 9 months [21].

Two experts who voted ‘Maybe’ (88.9% agreement) during the secondary voting expressed concerns due to a misunderstanding that annual anaemia screening for ages 1 to 5 involved mass screening regardless of risk factors for IDA. They were also concerned that using invasive screening methods may be excessive for young children with adequate solid food intake. Overall, the experts agreed that incorporating annual anaemia screening into regular healthcare or vaccination visits ensures early detection and timely intervention. This approach was seen as both practical and impactful, leveraging existing healthcare touchpoints to improve screening coverage. Some experts emphasised its cost‐effectiveness, while others highlighted its potential to reduce long‐term health complications, particularly in vulnerable populations. Considering the high prevalence of IDA in India and Southeast Asia, this consensus prioritises annual screening using noninvasive screening methods for all children between the ages of 1 and 5 years, which will be further deliberated in the following consensus statements.

### 4.4. Question 4: How to Screen Children for Anaemia?

#### 4.4.1. Consensus Statement 4: Screening for Anaemia Should Be Done Using a Noninvasive Haemoglobin Measurement Device

Previously, anaemia screening relied on invasive blood sampling of haemoglobin or haematocrit [25], but this has since evolved to include noninvasive methods using haemoglobin measurement devices such as Pronto (Masimo Corporation, California, United States) [26], Rad‐67 (Masimo Corporation, California, United States) [27], NBM‐200 (OrSense, Tel‐Aviv‐Yafo, Israel) [28] and Haemospect (MBR Optical Systems GmbH & Co. KG, Germany) [29]. These noninvasive devices provide pain‐free anaemia screening and deliver quick results at a low cost. They are particularly valuable as point‐of‐care testing modalities. A Thai study has demonstrated suboptimal uptake of anaemia screening among infants (76.1% uptake rate) primarily due to long waiting times for laboratory results [30].

Multiple studies have shown a good correlation between noninvasive testing, such as Masimo devices, and invasive testing in both adults and children [31–40]. Nonetheless, in instances where noninvasive testing devices are unavailable, validated and focused questionnaires should be considered in anaemia and IDA screening. The iron deficiency risk questionnaire includes domains such as dietary parameters and history related to birth, health, maternal or family background to detect the risk of IDA [41]. The food frequency questionnaire, on the other hand, is a validated tool to evaluate iron nutrition in infants by comparing 3‐day food record and iron status [42–44].

Four experts voted ‘Maybe’ (77.9% agreement) during the secondary voting, expressing concerns about the availability, accuracy and reliability of noninvasive haemoglobin measuring devices. One expert suggested that invasive screening methods should still be offered when noninvasive screening is unavailable to ensure comprehensive anaemia screening; the panel advised that noninvasive methods should be preferred if available. While some experts opined that blood tests may provide more accurate results, only validated noninvasive haemoglobin measurement devices should be preferred to address concerns about the accuracy and reliability of devices available in the market.

Nonetheless, 87.5% agreement was obtained from the primary expert group. Noninvasive haemoglobin measurement devices serve as a useful screening tool for anaemia in infants and children, avoiding the need for invasive testing. Other advantages of using noninvasive haemoglobin measurement include ease of execution without requiring highly specialised staff, suitability for large‐scale implementation at community level and being pain‐free making it less likely to be rejected by parents or caretakers. While more studies are underway to validate its accuracy, annual screening for anaemia in children should preferably be performed using verified and standardised noninvasive methods.

#### 4.4.2. Consensus Statement 5: Children Who Are Detected to Be Anaemic Using Noninvasive Screening Should Have Further Evaluation

Children suspected to be anaemic on noninvasive devices should undergo further evaluation using blood tests to confirm the diagnosis of anaemia. These tests should examine laboratory parameters such as complete blood count, iron profile (serum iron and ferritin), macrocytosis profile (vitamin B12 and folate), transferrin and transferrin saturation [45]. Once anaemia is confirmed, diagnostic tests for IDA should be performed, including a ferritin test, iron studies (serum iron, transferrin saturation and total iron‐binding capacity or transferrin concentration), mean cell volume, mean cell haemoglobin, red cell distribution width and measurement of soluble transferrin receptor [45]. In view of the substantial existing literature, the experts reached unanimous agreement (100%) that further evaluation is warranted for children found to be anaemic using noninvasive screening.

#### 4.4.3. Consensus Statement 6: As Thalassaemia Is Prevalent in India and Southeast Asia, Further Test(s) Should Be Performed Accordingly in Children Found to Have Anaemia

Children diagnosed with anaemia should also be tested for thalassaemia. In Malaysia, effective identification of screening of beta‐thalassaemia trait is achieved through screening 15‐ to 16‐year‐old students, premarital screening and screening of the relatives of known carriers [46]. In contrast, thalassaemia screenings in Thailand and Vietnam are performed prenatally [47, 48]. India offers optional screening programmes [49], whereas the Philippines implement a newborn bloodspot screening programme for haemoglobinopathies, including thalassaemia [50]. In addition, pilot programmes for thalassaemia screening and diagnosis have been introduced in Lao PDR with support from Thailand [51–53].

During the secondary voting, one expert who voted ‘Maybe’ (94.5% agreement) opined that thalassaemia screening should only be targeted at communities where thalassaemia is predominant. However, given the high prevalence of thalassaemia in India and Southeast Asia, it is reasonable to perform further testing to rule out thalassaemia upon anaemia diagnosis.

#### 4.4.4. Consensus Statement 7: IDA Is Prevalent in India and Southeast Asia; Hence, Iron Deficiency Should Be Ruled Out in Children Found to Have Anaemia

Iron deficiency affects up to 30% of children below age 5 in India and Southeast Asia, with a similar prevalence observed for IDA [54–60]. According to the WHO, *‘ferritin concentration is a good marker of iron stores and should be used to diagnosed iron deficiency in otherwise apparently healthy individuals’* without detectable diseases or conditions [61]. The recommended cut‐off values for ferritin are < 12 μg/L for infants and children under 5 years and < 15 μg/L for children aged 5 years and older, as well as adolescents and adults [61]. Ferritin testing to identify iron deficiency is usually performed alongside haemoglobin testing when assessing anaemia; other tests that may be used in conjunction include measures of inflammation (such as C‐reaction protein or α‐1 acid glycoprotein) and additional iron indices (such as soluble transferrin receptor) [61]. Given the burden of IDA in the region, the experts unanimously agreed (100%) that iron deficiency should emphasise the importance of assessing iron status, and iron deficiency should be ruled out in all children diagnosed with anaemia.

### 4.5. Question 5: What Strategies Can Be Implemented to Prevent and Manage IDA From Pregnancy Through Childhood? What Strategies and Government Policies Are in Place to Improve Iron Intake in Children?

#### 4.5.1. Consensus Statement 8: Nutritional Intervention, Particularly Food Fortification, Should Be Introduced to All Children to Reduce the Risk of IDA

Including iron‐fortified milk and foods in a child’s diet supports sufficient iron intake. The AAP recommends using iron‐fortified milk formula and iron‐containing foods, iron‐fortified cereals, for infants and toddlers starting at 4–6 months of age [16]. The CDC also supports that iron‐fortified infant formula can fulfil a child’s iron needs during infancy [62]. Iron fortification, combined with promoting a balanced diet, is an important and effective strategy to combat iron deficiency.

Milk is a useful vehicle for delivering micronutrients, including iron. Multiple systematic reviews support the use of iron‐fortified milk and foods to combat anaemia and iron deficiency, reporting improved haemoglobin levels and a reduction in anaemia cases [63, 64]. Focussing on low‐ or middle‐income countries, another systematic review and meta‐analysis determined that large‐scale food fortification with iron was associated with a modest yet meaningful increase in the haemoglobin concentration and a decline in anaemia and iron deficiency for preschool children, school‐age children and women of reproductive age [65]. Similarly, a more recent Cochrane review found that iron fortification of foods in infants may benefit low‐risk populations [66].

Fortified milk and foods have also shown improvement in iron status. A study done in New Zealand determined that iron‐fortified follow‐on milk and iron‐fortified partially modified cows’ milk resulted in significant increases in haemoglobin, iron saturation and mean cell volume, along with a reduction of IDA prevalence [67]. In addition, children who consumed iron‐supplemented milk formula had a lower probability of iron deficiency compared to those who received nonfortified cow’s milk [68]. Likewise, in a study involving 225 healthy nonanaemic children aged 12–20 months, serum ferritin levels increased significantly by 44% in 20 weeks in those consuming iron‐fortified milk [69].

Multi‐micronutrient fortification has also been shown to enhance growth and reduce the risk of IDA. Children consuming micronutrient‐fortified milk exhibited improvements in weight and height, accompanied with improvement in mean haemoglobin and serum ferritin levels, as well as an 88% lower risk of IDA compared to unfortified milk (*p* < 0.001) [70]. In Indonesia, a study of 5749 infants and young children aged 6–23 months found that fortified infant cereals reduced future production losses associated with IDA due to impaired physical activity, cognitive impairment and mortality by 43,000 disability‐adjusted life years, equating to USD 171 million [71]. Furthermore, fortification with candies containing 1 mg elemental iron/g, increased haemoglobin and serum ferritin concentrations and decreased anaemia prevalence in Indonesian children aged 4–6 years (*p* < 0.001 vs. baseline) [72].

Selection of iron compounds for food fortification should prioritise high bioavailability while minimising undesirable sensory alterations of food [73]. Water‐soluble iron compounds generally have the highest bioavailability due to their high solubility in gastric juices, making them the preferred choice for food fortification [73]. The type of iron provided by the food source is also crucial, as haem iron, found in animal sources, such as meat, poultry and seafood, is better absorbed than nonhaem iron from plant sources (37% vs. 5%) [74].

The absorption of iron is improved by ascorbic acid, with emerging evidence supporting a potential role for prebiotics. Ascorbic acid reduces ferric iron (Fe^3+^) to ferrous iron (Fe^2+^) and forms a chelate with iron to maintain its solubility, thus facilitating its transport through the intestinal microvilli to improve iron absorption [75, 76]. Formula milk fortified with iron sulphate and stabilised with maltodextrin and citric acid improved iron absorption by almost three‐fold (*p* < 0.001) as compared with cow’s milk fortified with iron [77]. Meanwhile, prebiotics such as fructo‐oligosaccharides, galacto‐oligosaccharides and *trans*‐galacto‐oligosaccharides promote the growth of beneficial gut bacteria that enhance iron absorption in infants [78–80]. Studies conducted in Kenya have also demonstrated the positive effects of prebiotics on iron absorption in infants, observed as increased iron absorption and reduced prevalence of anaemia [81, 82].

During the second voting, three experts voted ’Maybe’ (83.3% agreement) due to concerns over food fortification and iron overload. One expert noted that increased iron intake through food fortification may have adverse effects for individuals with haemoglobinopathies, leading to secondary iron overload. While this condition usually results from blood transfusion and iron therapy [83], it is less likely to occur from dietary iron absorption, as this process is typically regulated by the body’s current iron status to balance iron loss [84]. Another expert highlighted the critical importance of nutrient selection in food fortification, warning that improper choices could create a false sense of security regarding the nutritional value and health benefits of fortified foods. A similar concern was raised by another expert, who emphasised that food fortification, though a valuable strategy to address nutrient deficiencies, should not replace the need for a balanced and varied diet. Instead, food fortification should complement, not substitute, healthy dietary practices. Despite these valid points, the marked increase in iron requirements during a child’s growth years often necessitates a diet consistently high in meat and ascorbic acid–rich foods [85]. This dietary requirement is frequently met by commercially available products fortified with iron and ascorbic acid, usually consumed alongside fruit juices and solid foods that include meat, fish and vegetables [85].

Considering the available evidence, food and milk fortification with iron, ascorbic acid, prebiotics and other micronutrients is an important strategy for managing and preventing IDA. It is recommended that iron‐fortified foods be introduced starting at 4–6 months.

#### 4.5.2. Consensus Statement 9: Active Caregiver Education on IDA, Its Risks and Ways to Reduce the Likelihood of Developing IDA Is Recommended

Adequate education for parents or caregivers on IDA is crucial for improving iron status in children. A study involving mothers of children with anaemia aged 6–24 months found that an interventional health education programme enhanced mothers’ knowledge, leading to good dietary practices and notable increases in haemoglobin and haematocrit of their children [86]. This underscores the importance of actively educating parents or caregivers about IDA, its risks and effective strategies to prevent it, including promoting healthy behaviours and an iron‐rich diet within households, and is unanimously agreed upon (100%) by the experts.

#### 4.5.3. Consensus Statement 10: Improving Women’s Iron Status From Prepregnancy, During Pregnancy and Lactation Is Recommended to Reduce IDA Risk in Children

Iron status in women throughout various stages, including prepregnancy, pregnancy, postpregnancy and lactation, plays a crucial role in influencing the risk of IDA in children. Adequate iron store before pregnancy is essential to meet the increased iron demands to support the placenta and the growing foetus [87]. To improve pregnancy outcomes and enhance both maternal and infant health, strategies to address iron deficiency and anaemia should be integrated. This includes improving iron reserves and folate status in women through deworming initiatives and large‐scale fortification programmes of staple foods with iron and folic acid [88].

During pregnancy, maternal iron deficiency may reduce foetal iron stores, potentially affecting the infant’s iron level well into the first year of life [89]. Therefore, iron supplements are imperative for maintaining maternal iron status throughout pregnancy and the postpartum period, irrespective of their initial iron status [89]. A consensus statement on improving IDA management in Asia recommends that pregnant women with IDA should receive iron supplementation, as IDA increases the likelihood of maternal morbidity, preterm delivery and low birth weight [90].

Mothers’ iron status during pregnancy is closely linked to the prevalence of childhood anaemia. A study involving 167,017 children aged 6–59 months across six South and Southeast Asian countries found that childhood anaemia was significantly higher among mothers with anaemia, compared to nonanaemic mothers, with an AOR ranging from 1.44 to 1.71 [91]. In the same study, children in communities with a high percentage of maternal anaemia also had increased odds of childhood anaemia in all six countries, corresponding to an AOR of 1.21–1.72 [91]. Furthermore, a study of 295 nonanaemic pregnant women who received iron supplementation according to their haemoglobin levels before the 12^th^ week of gestation found that prenatal iron supplementation improved cognitive functioning in children by age 4 [92]. The WHO advises postpartum women to receive oral iron supplementation, with or without folic acid, for 6–12 weeks after delivery to lower the risk of anaemia in areas where gestational anaemia is a public health concern [93].

Breast milk contains small amounts of iron, and there is minimal evidence linking breast milk iron concentration and maternal iron status, except in cases of severe maternal anaemia [94, 95]. One expert disagreed (94.4% agreement) with the statement that improving maternal iron status during the lactation period would enhance the child’s iron status, arguing that iron content in breast milk is largely independent of maternal iron status or supplementation [96]. While it is true that increasing maternal iron stores may not substantially improve the iron content of breast milk if the mother has normal iron stores, preventing anaemia in lactating mothers remains imperative. The Food and Agriculture Organization and WHO recommend an absolute iron requirement of 1.15 mg/day for lactating women, which is lower than the iron requirement for nonlactating women (1.46 mg/day) [85]. Nonetheless, a study has shown that although breast milk iron content declines over the course of lactation, it remains relatively constant among anaemic women who received iron supplementation [97]. Therefore, maintaining good iron reserves during lactation through supplementation may ensure adequate iron transfer into breast milk.

#### 4.5.4. Consensus Statement 11: Deworming in Helminth‐Endemic Areas Is Encouraged to Curb Iron Loss Among Children

Deworming in children has been shown to prevent and improve childhood anaemia. A study conducted in Sub‐Saharan Africa with 50,075 children aged 6–59 months (61.8% prevalence of anaemia) found that children who did not receive deworming medication had increased odds of being anaemic (AOR: 1.11; 95% CI 1.07–1.16) [98]. Another analysis of five cross‐sectional surveys in Bangladesh, involving 9948 children aged 6–59 months, reported that those who received deworming and effective micronutrient powder supplementation were 30% less likely to be anaemic (AOR: 0.7; 95% CI 0.52–0.94) compared to children who did not receive these interventions [99]. A systematic review and meta‐analysis involving more than one million school children in Tanzania, India, Nigeria, Thailand, Zanzibar and Vietnam reported an overall improvement in haemoglobin levels by 1.62 g/dL (95% CI 1.01–2.25) after deworming [100]. In addition, a Thai study involving 182 hookworm‐positive schoolchildren aged 5–17 years found that deworming improved haemoglobin, haematocrit, total protein and albumin levels within 2 months to levels comparable to a control of 57 helminth‐free children, with sustained improvements even after a year [101].

All experts collectively agreed (100%) that public health programmes should incorporate deworming alongside nutrient supplementation as part of a comprehensive strategy to reduce childhood anaemia. The WHO recommends deworming children at least once a year, or twice a year, if worm infection is particularly common. Deworming pills can be administered to children from 1 year of age, regardless of their size or weight [102].

#### 4.5.5. Consensus Statement 12: Children Found to Have IDA Should Receive Iron Treatment in Addition to Other Measures to Prevent or Treat IDA

Children with IDA have been shown to benefit from iron treatment. Common oral treatments include ferrous fumarate, ferrous sulphate, ferrous gluconate and ferrous bisglycinate, while common intravenous treatments include iron sucrose, iron dextran, ferric gluconate, ferumoxytol, ferric carboxymaltose and ferric derisomaltose [103].

Oral iron supplementation is an affordable yet effective treatment for IDA in stable outpatients [104]. Meanwhile, intravenous iron therapy is effective for raising haemoglobin levels in children with IDA who are poorly compliant or unresponsive with oral iron therapy or diagnosed with severe anaemia [105]. Intravenous iron transfusions are also indicated for premature newborns, patients unresponsive to oral iron treatment, haemodialysis‐dependent patients and patients with chronic diseases such as renal failure, bowel diseases and epidermolysis bullosa [106, 107].

A study involving 90 Pakistani children with IDA aged 12–60 months who did not respond adequately to oral iron therapy reported an increase in mean haemoglobin after receiving intravenous iron in two divided doses over two consecutive days (mean haemoglobin at baseline 5.9 ± 1.3 g/dL, at 2 weeks 8.38 ± 1.09 g/dL and at 4 weeks 9.74 ± 0.88 g/dL; *p* < 0.05) [108]. Another study involving 44 Turkish children aged 0.5–18.5 years with IDA found that the treatments with intravenous iron sucrose or ferric carboxymaltose increased haemoglobin, mean corpuscular volume, mean corpuscular haemoglobin, red‐cell distribution width and serum ferritin levels, while decreasing platelet count [105].

Children diagnosed with IDA have shown meaningful improvements in iron status, highlighting the importance of iron treatment for this patient group. One expert voted ‘Maybe’ (94.4% agreement) emphasised that the IDA severity should be assessed before deciding on the treatment plan. The appropriate treatment route, whether oral or intravenous, should be decided based on the severity of IDA, patient compliance and responsiveness towards the respective treatment.

### 4.6. Strengths and Limitations

This consensus featured several strengths. Firstly, it provides valuable insights and perspectives from a panel of experts from diverse specialisations, including paediatrics, nutrition, family medicine, public health and obstetrics and gynaecology. The experts examined clinical data and current practices, producing practical recommendations for screening, managing and preventing IDA among Indian and Southeast Asian children from ages 1–5 years. Secondly, even though developed through a consensus approach, these statements are evidence‐based, drawn from the medical literature and current guidelines on IDA diagnosis and management, with a specific focus on children in India and Southeast Asia.

However, there are a few limitations to the consensus. Despite inputs from leading experts in India and Southeast Asia to develop these consensus statements, not every Southeast Asian country was represented in the working group. Furthermore, anonymous live voting and the lack of virtual or face‐to‐face discussions following the secondary expert group’s Delphi round may have hampered panellists from discussing relevant information and clarifying grounds for disagreement.

## 5. Conclusion

Children in India and Southeast Asia continue to be affected by IDA. This expert consensus was developed with the goals of enhancing awareness, providing specific recommendations for screening and management of children at risk of IDA and supporting the prevention of IDA in children. The final 12 consensus statements addressed topics such as the recommended age for anaemia screening, the use of noninvasive haemoglobin measurement devices, further evaluations to rule out thalassaemia and IDA in children with anaemia, as well as suggested interventions to reduce the risk of IDA. These statements are intended to guide policymakers and health practitioners in decision‐making related to the screening, treatment and prevention of IDA among children in these countries.

## Disclosure

All authors have approved the final manuscript and accept responsibility for its accuracy and integrity. All contents were reviewed and approved by the authors, who take full responsibility for the manuscript.

## Conflicts of Interest

M.Y.J., H.J.J.M., S.W.T., K.A., S.S., K.P. and A.P. provide consultancy to Danone. M.Y.J., K.A. and S.S. have received research grant from Danone. M.Y.J., H.J.J.M., S.W.T., K.A., L.P., S.S., K.P. and A.P. have received speaker’s fees from Danone. R.B. and S.K.A. declare no conflicts of interest.

## Author Contributions

M.Y.J., H.J.J.M., S.W.T., K.A., L.P., S.S., K.P., A.P., R.B. and S.K.A. conceptualised and prepared the original draft, which was subsequently reviewed and edited by all authors.

## Funding

The advisory board logistic organisation and the development of this article were supported by Danone. No authors have received honorarium or funding for the development of this consensus.

## Data Availability

The data that support the findings of this study are available from the corresponding author upon reasonable request.

## References

[1] Adam H. , Screening for Anemia, Pediatric Care Online. (2020) 10.1542/aap.ppcqr.396021.

[2] A]lali S. , Brousse V. , Sacri A. S. , Chalumeau M. , and de Montalembert M. , Anemia in Children: Prevalence, Causes, Diagnostic Work-Up, and Long-Term Consequences, Expert Review of Hematology. (2017) 10, no. 11, 1023–1028, 10.1080/17474086.2017.1354696, 2-s2.0-85032662766. 29023171

[3] World Health Organization , Guideline on Haemoglobin Cutoffs to Define Anaemia in Individuals and Populations, 2024, https://www.who.int/publications/i/item/9789240088542. 38530913

[4] World Health Organization , Prevalence of Anaemia in Children Aged 6–59 Months (%), 2021, https://www.who.int/data/gho/data/indicators/indicator-details/gho/prevalence-of-anaemia-in-children-under-5-years.

[5] The Royal Children’s Hospital , Clinical Practice Guidelines: Iron Deficiency, 2023, https://www.rch.org.au/clinicalguide/guideline_index/Iron_deficiency/.

[6] Bhatnagar R. S. and Padilla-Zakour O. I. , Plant-Based Dietary Practices and Socioeconomic Factors That Influence Anemia in India, Nutrients. (2021) 13, no. 10, 10.3390/nu13103538. PMC8537570 34684539

[7] Alnaz A. R. M. , Darlan D. M. , Andriyani Y. , and Lubis R. , Hemoglobin Level and Risk of Anemia in Soil-Transmitted Helminths Infections Among Children: A Systematic Review and Meta-Analysis, Open Access Macedonian Journal of Medical Sciences. (2022) 10, 355–363, 10.3889/oamjms.2022.8974.

[8] Gallagher P. G. , Anemia in the Pediatric Patient, Blood. (2022) 140, no. 6, 571–593, 10.1182/blood.2020006479. 35213686 PMC9373018

[9] Kotwal A. , Iron Deficiency Anaemia Among Children in South East Asia: Determinants, Importance, Prevention and Control Strategies, Current Medicine Research and Practice. (2016) 6, no. 3, 117–122, 10.1016/j.cmrp.2016.05.009.

[10] Krämer M. , Kumar S. , and Vollmer S. , Anemia, Diet, and Cognitive Development: Impact of Health Information on Diet Quality and Child Nutrition in Rural India, Journal of Economic Behavior & Organization. (2021) 190, 495–523, 10.1016/j.jebo.2021.06.043.

[11] Cappellini M. D. , Musallam K. M. , and Taher A. T. , Iron Deficiency Anaemia Revisited, Journal of Internal Medicine. (2020) 287, no. 2, 153–170, 10.1111/joim.13004. 31665543

[12] Grime M. M. and Wright G. , Delphi Method, Wiley StatsRef: Statistics Reference Online, 2016, 1–6, 10.1002/9781118445112.stat07879.

[13] CareSearch , PipoS/PIPOH, https://www.caresearch.com.au/Portals/20/Documents/Evidence/Forms/PIPOS_PIPOH-form-fillable.pdf.

[14] De Meyer D. , Kottner J. , Beele H. et al., Delphi Procedure in Core Outcome Set Development: Rating Scale and Consensus Criteria Determined Outcome Selection, Journal of Clinical Epidemiology. (2019) 111, 23–31, 10.1016/j.jclinepi.2019.03.011, 2-s2.0-85064259913. 30922885

[15] World Health Organization , Archived: Iron Deficiency Anaemia: Assessment, Prevention and Control, 2001, https://cdn.who.int/media/docs/default-source/2021-dha-docs/ida_assessment_prevention_control.pdf.

[16] Baker R. D. , Greer F. R. , and Committee on Nutrition American Academy of Pediatrics , Diagnosis and Prevention of Iron Deficiency and Iron-Deficiency Anemia in Infants and Young Children (0-3 Years of Age), Pediatrics. (2010) 126, no. 5, 1040–1050, 10.1542/peds.2010-2576, 2-s2.0-78049422747. 20923825

[17] Janus J. and Moerschel S. K. , Evaluation of Anemia in Children, American Family Physician. (2010) 81, no. 12, 1462–1471. 20540485

[18] Ministry of Health and Family Welfare India , Guidelines for Control of Iron Deficiency Anaemia, 2013, https://nhm.gov.in/images/pdf/programmes/wifs/guidelines/Guidelines_for_Control_of_Iron_Deficiency_Anaemia.pdf.

[19] National Pediatric Hospital Cambodia , Nph′s Practical Guideline Version 1: Iron Deficiency Anemia in Children, 2020.

[20] Sekartini R. , Widjaja N. A. , Mutu Manikam N. R. , Jo J. , Basrowi R. W. , and Dilantika C. , Iron-Deficiency Anemia: Indonesia’s Striving, Asia Pac J Paediatr Child Health. (2022) 5, 3–16, https://apjpch.com/pdfs/2297hJz080011.pdf.

[21] The Royal College of Pediatricians of Thailand, & Paediatric Society of Thailand , Guideline in Child Health Supervision, 2021, https://www.thaipediatrics.org/?p=866.

[22] Sungkar A. , Bardosono S. , Irwinda R. et al., A Life Course Approach to the Prevention of Iron Deficiency Anemia in Indonesia, Nutrients. (2022) 14, no. 2, 10.3390/nu14020277. PMC8780595 35057458

[23] Kohli-Kumar M. , Screening for Anemia in Children: AAP Recommendations--A Critique, Pediatrics. (2001) 108, no. 3, 10.1542/peds.108.3.e56. 11533374

[24] Xin Q. Q. , Chen B. W. , Yin D. L. et al., Prevalence of Anemia and Its Risk Factors Among Children Under 36 Months Old in China, Journal of Tropical Pediatrics. (2017) 63, no. 1, 36–42, 10.1093/tropej/fmw049, 2-s2.0-85014673297. 27543970

[25] Siu A. L. and US Preventive Services Task Force , Screening for Iron Deficiency Anemia in Young Children: UspsTF Recommendation Statement, Pediatrics. (2015) 136, no. 4, 746–752, 10.1542/peds.2015-2567, 2-s2.0-84942849018. 26347426

[26] Masimo , Pronto® Pulse Co-Oximeter®, 2018, https://professional.masimo.com/products/monitors/spot-check/pronto/.

[27] Masimo , Rad-67® Pulse CO-Oximeter®, 2023, https://www.masimo.com/products/monitors/spot-check/rad-67/.

[28] Orsense Ltd , Orsense NBM-200 Non-Invasive Hemoglobin Device Implemented by Children’s Hospital Los Angeles, 2019, https://www.prnewswire.com/news-releases/orsense-nbm-200-non-invasive-hemoglobin-device-implemented-by-childrens-hospital-los-angeles-300910682.html.

[29] Attikouris Medical , Hemoglobin Device: Haemospect by MBR, http://www.attikourismedical.com/product/diagnostic/haemospect-device/.

[30] Chienwittayakun K. , Suteerojntrakool O. , Techavichit P. , Bongsebandhu-Phubhakdi C. , Subchartanan J. , and Tempark T. , Adherence to Screening for Anemia in 9-Month-Old Full-Term Infants in Bangkok, Thailand, Journal of Tropical Pediatrics. (2021) 67, no. 3, 10.1093/tropej/fmaa054. 33057692

[31] Amano I. and Murakami A. , Use of Non-Invasive Total Hemoglobin Measurement as a Screening Tool for Anemia in Children, Pediatrics International. (2013) 55, no. 6, 803–805, 10.1111/ped.12236, 2-s2.0-84890485688. 24330295

[32] Arai Y. , Shoji H. , Awata K. , Inage E. , Ikuse T. , and Shimizu T. , Evaluation of the Use of Non-Invasive Hemoglobin Measurement in Early Childhood, Pediatric Research. (2023) 93, no. 4, 1036–1040, 10.1038/s41390-022-02204-7. 35906313

[33] Hiscock R. , Kumar D. , and Simmons S. W. , Systematic Review and Meta-Analysis of Method Comparison Studies of Masimo Pulse co-Oximeters (Radical-7™ or Pronto-7™) and HemoCue® Absorption Spectrometers (B-Hemoglobin or 201+) With Laboratory Haemoglobin Estimation, Anaesthesia & Intensive Care. (2015) 43, no. 3, 341–350, 10.1177/0310057x1504300310. 25943608

[34] Hsu D. P. , French A. J. , Madson S. L. , Palmer J. M. , and Gidvani-Diaz V. , Evaluation of a Noninvasive Hemoglobin Measurement Device to Screen for Anemia in Infancy, Maternal and Child Health Journal. (2016) 20, no. 4, 827–832, 10.1007/s10995-015-1913-9, 2-s2.0-84961113379. 26702617

[35] Panda S. K. , Mishra A. , and Jena P. K. , Agreement Between Noninvasive Hemoglobin and Laboratory Hemoglobin Measurements in Neonates: A Systematic Review and Meta-Analysis, Neonatology. (2023) 120, no. 1, 24–32, 10.1159/000526100. 36450265

[36] Payton M. , Comparison Noninvasive Hemoglobin and Invasive Spun Hematocrit Testing in WIC Participants, https://professional.masimo.com/siteassets/us/documents/pdf/clinical-evidence/sphb/8260b_poster_wic.pdf.

[37] Ramaswamy G. , Vohra K. , Yadav K. et al., Point-Of-Care Testing Using Invasive and Non-Invasive Hemoglobinometers: Reliable and Valid Method for Estimation of Hemoglobin Among Children 6-59 Months, Journal of Tropical Pediatrics. (2021) 67, no. 1, 10.1093/tropej/fmaa111. 33367788

[38] Shah N. , Osea E. A. , and Martinez G. J. , Accuracy of Noninvasive Hemoglobin and Invasive Point-Of-Care Hemoglobin Testing Compared With a Laboratory Analyzer, International Journal of Laboratory Hematology. (2014) 36, no. 1, 56–61, 10.1111/ijlh.12118, 2-s2.0-84892483949. 23809685 PMC4232003

[39] Shamah Levy T. , Méndez-Gómez-Humarán I. , Morales Ruán M. D. C. , Martinez Tapia B. , Villalpando Hernández S. , and Hernández Ávila M. , Validation of Masimo Pronto 7 and HemoCue 201 for Hemoglobin Determination in Children From 1 to 5 Years of Age, Plos One. (2017) 12, no. 2, 10.1371/journal.pone.0170990, 2-s2.0-85011966460. PMC5295714 28170445

[40] Wittenmeier E. , Bellosevich S. , Mauff S. et al., Comparison of the Gold Standard of Hemoglobin Measurement With the Clinical Standard (BGA) and Noninvasive Hemoglobin Measurement (SpHb) in Small Children: A Prospective Diagnostic Observational Study, Paediatric Anaesthesia. (2015) 25, no. 10, 1046–1053, 10.1111/pan.12683, 2-s2.0-84941059586. 26179143

[41] Bogen D. L. , Duggan A. K. , Dover G. J. , and Wilson M. H. , Screening for Iron Deficiency Anemia by Dietary History in a High-Risk Population, Pediatrics. (2000) 105, no. 6, 1254–1259, 10.1542/peds.105.6.1254, 2-s2.0-0034128779. 10835066

[42] Nurul-Fadhilah A. , Teo P. S. , and Foo L. H. , Validity and Reproducibility of a Food Frequency Questionnaire (FFQ) for Dietary Assessment in Malay Adolescents in Malaysia, Asia Pacific Journal of Clinical Nutrition. (2012) 21, no. 1, 97–103. 22374566

[43] Sugianto R. , Chan M. J. , Wong S. F. et al., Evaluation of a Quantitative Food Frequency Questionnaire for 5-Year-Old Children in an Asian Population, Journal of the Academy of Nutrition and Dietetics. (2020) 120, no. 3, 437–444, 10.1016/j.jand.2019.09.021. 31866358 PMC7039700

[44] Williams P. L. and Innis S. M. , Food Frequency Questionnaire for Assessing Infant Iron Nutrition, Canadian Journal of Dietetic Practice and Research. (2005) 66, no. 3, 176–182, 10.3148/66.3.2005.176, 2-s2.0-24744468384. 16159411

[45] Bouri S. and Martin J. , Investigation of Iron Deficiency Anaemia, Clinical Medicine. (2018) 18, no. 3, 242–244, 10.7861/clinmedicine.18-3-242, 2-s2.0-85048416093. 29858435 PMC6334077

[46] Mat M. A. C. , Yaacob L. H. , and Zakaria R. , Parental Knowledge on Thalassaemia and Factors Associated With Refusal to Screen Their Children, Malaysian Journal of Medical Sciences. (2020) 27, no. 1, 124–133, 10.21315/mjms2020.27.1.13. PMC7053551 32158352

[47] Tran D. C. , Dang A. L. , Hoang T. N. L. et al., Prevalence of Thalassemia in the Vietnamese Population and Building a Clinical Decision Support System for Prenatal Screening for Thalassemia, Mediterranean Journal of Hematology and Infectious Diseases. (2023) 15, no. 1, 10.4084/MJHID.2023.026. PMC10171208 37180206

[48] Xu J. Z. , Foe M. , Tanongsaksakul W. et al., Identification of Optimal Thalassemia Screening Strategies for Migrant Populations in Thailand Using a Qualitative Approach, BMC Public Health. (2021) 21, no. 1, 10.1186/s12889-021-11831-4. PMC8495975 34615515

[49] Thiyagarajan A. , Bhattacharya S. , Sharma N. , Srivastava A. , and Dhar D. K. , Need for a Universal Thalassemia Screening Programme in India? A Public Health Perspective, Journal of Family Medicine and Primary Care. (2019) 8, no. 5, 1528–1532, 10.4103/jfmpc.jfmpc_90_19. PMC6559078 31198708

[50] Padilla C. D. , Therrell B. L.Jr., Alcausin M. et al., Successful Implementation of Newborn Screening for Hemoglobin Disorders in the Philippines, International Journal of Neonatal Screening. (2021) 7, no. 2, 10.3390/ijns7020030. PMC8293152 34204320

[51] Savongsy O. , Fucharoen S. , Fucharoen G. , Sanchaisuriya K. , and Sae-Ung N. , Thalassemia and Hemoglobinopathies in Pregnant Lao Women: Carrier Screening, Prevalence and Molecular Basis, Annals of Hematology. (2008) 87, no. 8, 647–654, 10.1007/s00277-008-0490-Z, 2-s2.0-46949111212. 18414862

[52] Somphanthabansouk K. , Sanchaisuriya K. , Vidamaly V. et al., Pilot Screening Program for Thalassemia Carriers at Community Level in Lao People’s Democratic Republic *Southeast Asian* , Journal of Tropical Medicine. (2017) 48, no. 5, 1083–1092.

[53] Wongprachum K. , Sanchaisuriya K. , Vidamaly V. et al., Pilot Screening Program for Thalassemia in a Country With Limited Resources: A Collaboration Model Between Close Neighboring Countries *Southeast Asian* , Southeast Asian Journal of Tropical Medicine & Public Health. (2016) 47, no. 5, 1040–1047. 29620818

[54] Alexandra K. , Malnutrition in the Philippines: Perhaps a Double Burden?, 2009.

[55] Keokenchanh S. , Kounnavong S. , Midorikawa K. et al., Prevalence of Anemia and Its Associated Factors Among Children Aged 6-59 Months in the Lao People’s Democratic Republic: A Multilevel Analysis, Plos One. (2021) 16, no. 3, 10.1371/journal.pone.0248969. PMC7993607 33765048

[56] Kounnavong S. , Sunahara T. , Hashizume M. et al., Anemia and Related Factors in Preschool Children in the Southern Rural Lao People’s Democratic Republic, Tropical Medicine and Health. (2011) 39, no. 4, 95–103, 10.2149/tmh.2011-13, 2-s2.0-84863144998. 22438698 PMC3289278

[57] Kounnavong S. , Vonglokham M. , Kounnavong T. , Kwadwo D. D. , and Essink D. R. , Anaemia Among Adolescents: Assessing a Public Health Concern in Lao PDR, Global Health Action. (2020) 13, no. sup2, 10.1080/16549716.2020.1786997. PMC7480417 32741354

[58] Ministry of Health and Family Welfare India , Comprehensive National Nutrition Survey, 2023, https://knowledge.unicef.org/resource/comprehensive-national-nutrition-survey-2016-2018.

[59] Nik Shanita S. , Siti Hanisa A. , Noor Afifah A. R. et al., Prevalence of Anaemia and Iron Deficiency Among Primary Schoolchildren in Malaysia, International Journal of Environmental Research and Public Health. (2018) 15, no. 11, 10.3390/ijerph15112332, 2-s2.0-85056113089. PMC6266561 30360488

[60] Rohner F. , Woodruff B. A. , Aaron G. J. et al., Infant and Young Child Feeding Practices in Urban Philippines and Their Associations With Stunting, Anemia, and Deficiencies of Iron and Vitamin A, Food and Nutrition Bulletin. (2013) 34, no. 2 Suppl, S17–S34, 10.1177/15648265130342s104. 24049993

[61] World Health Organization , WHO Guideline on Use of Ferritin Concentrations to Assess Iron Status in Individuals and Populations, 2020, https://www.who.int/publications/i/item/9789240000124. 33909381

[62] Centers for Disease Control and Prevention , Iron: Infant and Toddler Nutrition, 2024, https://www.cdc.gov/infant-toddler-nutrition/vitamins-minerals/iron.html.

[63] Eichler K. , Wieser S. , Rüthemann I. , and Brügger U. , Effects of Micronutrient Fortified Milk and Cereal Food for Infants and Children: A Systematic Review, BMC Public Health. (2012) 12, 10.1186/1471-2458-12-506, 2-s2.0-84863471610. PMC3444335 22770558

[64] Vohra K. , Mittal M. , Verma A. , Keshri A. , Dhasmana A. , and al e. , Effect of Iron Fortified Milk and Milk Products on Anemia Status Among the Population – a Review, Indian Journal of Nutrition. (2021) 8, no. 1.

[65] Keats E. C. , Neufeld L. M. , Garrett G. S. , Mbuya M. N. N. , and Bhutta Z. A. , Improved Micronutrient Status and Health Outcomes in low- and Middle-Income Countries Following Large-Scale Fortification: Evidence From a Systematic Review and Meta-Analysis, American Journal of Clinical Nutrition. (2019) 109, no. 6, 1696–1708, 10.1093/ajcn/nqz023, 2-s2.0-85067269338. 30997493 PMC6537942

[66] da Silva Lopes K. , Yamaji N. , Rahman M. O. et al., Nutrition-Specific Interventions for Preventing and Controlling Anaemia Throughout the Life Cycle: An Overview of Systematic Reviews, Cochrane Database of Systematic Reviews. (2021) 9, no. 9, 10.1002/14651858.CD013092.pub2. PMC8464655 34564844

[67] Wall C. R. , Grant C. C. , Taua N. , Wilson C. , and Thompson J. M. , Milk Versus Medicine for the Treatment of Iron Deficiency Anaemia in Hospitalised Infants, Archives of Disease in Childhood. (2005) 90, no. 10, 1033–1038, 10.1136/adc.2004.067876, 2-s2.0-26044478725. 15956047 PMC1720139

[68] Akkermans M. D. , Eussen S. R. , van der Horst-Graat J. M. , van Elburg R. M. , van Goudoever J. B. , and Brus F. , A Micronutrient-Fortified Young-Child Formula Improves the Iron and Vitamin D Status of Healthy Young European Children: A Randomized, Double-Blind Controlled Trial, American Journal of Clinical Nutrition. (2017) 105, no. 2, 391–399, 10.3945/ajcn.116.136143, 2-s2.0-85011702291. 28052885

[69] Szymlek-Gay E. A. , Ferguson E. L. , Heath A. L. , Gray A. R. , and Gibson R. S. , Food-Based Strategies Improve Iron Status in Toddlers: A Randomized Controlled Trial12, American Journal of Clinical Nutrition. (2009) 90, no. 6, 1541–1551, 10.3945/ajcn.2009.27588, 2-s2.0-72749085313. 19828711

[70] Sazawal S. , Dhingra U. , Dhingra P. et al., Micronutrient Fortified Milk Improves Iron Status, Anemia and Growth Among Children 1-4 Years: A Double Masked, Randomized, Controlled Trial, Plos One. (2010) 5, no. 8, 10.1371/journal.pone.0012167, 2-s2.0-77957861492. PMC2921413 20730057

[71] Prieto-Patron A. , Detzel P. , Ramayulis R. , Sudikno I. , Wibowo Y. , and Wibowo Y. , Impact of Fortified Infant Cereals on the Burden of Iron Deficiency Anemia in 6- to 23-Month-Old Indonesian Infants and Young Children: A Health Economic Simulation Model, International Journal of Environmental Research and Public Health. (2022) 19, no. 9, 10.3390/ijerph19095416. PMC9105951 35564811

[72] Sari M. , Bloem M. W. , de Pee S. , Schultink W. J. , and Sastroamidjojo S. , Effect of Iron-Fortified Candies on the Iron Status of Children Aged 4-6 Y in East Jakarta, Indonesia, American Journal of Clinical Nutrition. (2001) 73, no. 6, 1034–1039, 10.1093/ajcn/73.6.1034. 11382656

[73] World Health Organization and Food and Agricultural Organization of the United Nation , Guidelines on Food Fortification With Micronutrients, 2006, https://www.who.int/publications/i/item/9241594012.

[74] Piskin E. , Cianciosi D. , Gulec S. , Tomas M. , and Capanoglu E. , Iron Absorption: Factors, Limitations, and Improvement Methods, ACS Omega. (2022) 7, no. 24, 20441–20456, 10.1021/acsomega.2c01833. 35755397 PMC9219084

[75] Lynch S. R. and Stoltzfus R. J. , Iron and Ascorbic Acid: Proposed Fortification Levels and Recommended Iron Compounds, Journal of Nutrition. (2003) 133, no. 9, 2978s–84S, 10.1093/jn/133.9.2978S. 12949396

[76] Subroto E. , Indiarto R. , and Andoyo R. , Bioavailability of Iron and Its Potential to Improve the Immune System and Ward off CoviD-19: A Review, Food Research. (2023) .

[77] Pizarro F. , Olivares M. , Maciero E. , Krasnoff G. , Cócaro N. , and Gaitan D. , Iron Absorption From Two Milk Formulas Fortified With Iron Sulfate Stabilized With Maltodextrin and Citric Acid, Nutrients. (2015) 7, no. 11, 8952–8959, 10.3390/nu7115448, 2-s2.0-84946062060. 26529007 PMC4663576

[78] Christides T. , Ganis J. C. , and Sharp P. A. , In Vitro Assessment of Iron Availability From Commercial Young Child Formulae Supplemented With Prebiotics, European Journal of Nutrition. (2018) 57, no. 2, 669–678, 10.1007/s00394-016-1353-3, 2-s2.0-85003875085. 27942845 PMC5845627

[79] Davani-Davari D. , Negahdaripour M. , Karimzadeh I. et al., Prebiotics: Definition, Types, Sources, Mechanisms, and Clinical Applications, Foods. (2019) 8, no. 3, 10.3390/foods8030092, 2-s2.0-85063289431. PMC6463098 30857316

[80] Zakrzewska Z. , Zawartka A. , Schab M. et al., Prebiotics, Probiotics, and Postbiotics in the Prevention and Treatment of Anemia, Microorganisms. (2022) 10, no. 7, 10.3390/microorganisms10071330. PMC9317605 35889049

[81] Paganini D. , Uyoga M. A. , Cercamondi C. I. et al., Consumption of Galacto-Oligosaccharides Increases Iron Absorption From a Micronutrient Powder Containing Ferrous Fumarate and Sodium Iron Edta: A Stable-Isotope Study in Kenyan Infants, American Journal of Clinical Nutrition. (2017) 106, no. 4, 1020–1031, 10.3945/ajcn.116.145060, 2-s2.0-85031696240. 28814396

[82] Paganini D. , Uyoga M. A. , Kortman G. A. M. et al., Prebiotic Galacto-Oligosaccharides Mitigate the Adverse Effects of Iron Fortification on the Gut Microbiome: A Randomised Controlled Study in Kenyan Infants, Gut. (2017) 66, no. 11, 1956–1967, 10.1136/gutjnl-2017-314418, 2-s2.0-85030999991. 28774885

[83] Omena J. , Curioni C. , Cople-Rodrigues C. D. S. , and Citelli M. , The Effect of Food and Nutrients on Iron Overload: What Do We Know so Far?, European Journal of Clinical Nutrition. (2021) 75, no. 12, 1771–1780, 10.1038/s41430-021-00887-5. 33712721

[84] Radford-Smith D. E. , Powell E. E. , and Powell L. W. , Haemochromatosis: A Clinical Update for the Practising Physician, Internal Medicine Journal. (2018) 48, no. 5, 509–516, 10.1111/imj.13784, 2-s2.0-85046372058. 29722188

[85] World Health Organization and Food and Agricultural Organization of the United Nation , Vitamin and Mineral Requirements in Human Nutrition, 2004, https://www.who.int/publications/i/item/9241546123.

[86] Hassan A. E. , Kamal M. M. , Fetohy E. M. , and Turky G. M. , Health Education Program for Mothers of Children Suffering From Iron Deficiency Anemia in United Arab Emirates, Journal of the Egyptian Public Health Association. (2005) 80, no. 5-6, 525–545. 17187741

[87] Milman N. , Taylor C. L. , Merkel J. , and Brannon P. M. , Iron Status in Pregnant Women and Women of Reproductive Age in Europe, American Journal of Clinical Nutrition. (2017) 106, no. Suppl 6, 1655s–1662s, 10.3945/ajcn.117.156000, 2-s2.0-85037650195. 29070543 PMC5701710

[88] World Health Organization , WHO Guidelines Approved by the Guidelines Review Committee, 2009.

[89] Allen L. H. , Anemia and Iron Deficiency: Effects on Pregnancy Outcome, American Journal of Clinical Nutrition. (2000) 71, no. 5 Suppl, 1280s–4S, 10.1093/ajcn/71.5.1280s. 10799402

[90] Leung T. W. , Damodaran P. , Torres R. et al., Expert Consensus on Improving Iron Deficiency Anemia Management in Obstetrics and Gynecology in Asia, International Journal of Gynaecology & Obstetrics. (2023) 163, no. 2, 495–509, 10.1002/ijgo.14804. 37096333

[91] Sunuwar D. R. , Singh D. R. , Pradhan P. M. S. et al., Factors Associated With Anemia Among Children in South and Southeast Asia: A Multilevel Analysis, BMC Public Health. (2023) 23, no. 1, 10.1186/s12889-023-15265-Y. PMC9933407 36793012

[92] Iglesias-Vázquez L. , Voltas N. , Hernández-Martínez C. et al., Importance of Maternal Iron Status on the Improvement of Cognitive Function in Children After Prenatal Iron Supplementation, American Journal of Preventive Medicine. (2023) 65, no. 3, 395–405, 10.1016/j.amepre.2023.02.006. 36906495

[93] World Health Organization , Guideline: Iron Supplementation in Postpartum Women, 2016, https://www.who.int/publications/i/item/9789241549585. 27583315

[94] Centers for Disease Control and Prevention , Iron, 2024, https://www.cdc.gov/breastfeeding-special-circumstances/hcp/diet-micronutrients/iron.html.

[95] Holm C. , Thomsen L. L. , Norgaard A. , Markova V. , Michaelsen K. F. , and Langhoff-Roos J. , Iron Concentration in Breast Milk Normalised Within One Week of a Single High-Dose Infusion of Iron Isomaltoside in Randomised Controlled Trial, Acta Paediatrica. (2017) 106, no. 2, 256–260, 10.1111/apa.13681, 2-s2.0-85007106735. 27883237

[96] Domellöf M. , Lönnerdal B. , Dewey K. G. , Cohen R. J. , and Hernell O. , Iron, Zinc, and Copper Concentrations in Breast Milk Are Independent of Maternal Mineral Status, American Journal of Clinical Nutrition. (2004) 79, no. 1, 111–115, 10.1093/ajcn/79.1.111. 14684406

[97] Bugtong-Belino P. , Oral Iron Supplementation: Effects on Maternal and Infant Iron Status and on Breastmilk Iron Concentration, 2010, University of the Philippines Los Baños, https://www.uplbgraduateschool.org/img/thesisfiles/Oral%20Iron%20Supplementation_Effects%20on%20Maternal%20and%20Infant%20Iron%20Status%20by%20Pelin%20B.%20Belino.pdf.

[98] Bauleni A. , Tiruneh F. N. , Mwenyenkulu T. E. et al., Effects of Deworming Medication on Anaemia Among Children Aged 6-59 Months in Sub-Saharan Africa, Parasites & Vectors. (2022) 15, no. 1, 10.1186/s13071-021-05123-4. PMC8753868 35016722

[99] Sarma H. , Wangdi K. , Tariqujjaman M. et al., The Effects of Deworming and Multiple Micronutrients on Anaemia in Preschool Children in Bangladesh: Analysis of Five Cross-Sectional Surveys, Nutrients. (2021) 14, no. 1, https://www.mdpi.com/2072-6643/14/1/150, 10.3390/nu14010150. PMC8746749 35011023

[100] Girum T. and Wasie A. , The Effect of Deworming School Children on Anemia Prevalence: A Systematic Review and Meta-Analysis, The Open Nursing Journal. (2018) 12, 155–161, 10.2174/1874434601812010155, 2-s2.0-85053079248. 30197721 PMC6110060

[101] Watthanakulpanich D. , Maipanich W. , Pubampen S. et al., Impact of Hookworm Deworming on Anemia and Nutritional Status Among Children in Thailand, Southeast Asian Journal of Tropical Medicine and Public Health. (2011) 42, no. 4, 782–792. 22299460

[102] World Health Organization and World Bank Group , School Deworming at a Glance, 2003, https://www.who.int/publications/i/item/School-deworming-at-a-glance-2003.

[103] Deloughery T. G. , Jackson C. S. , Ko C. W. , and Rockey D. C. , AGA Clinical Practice Update on Management of Iron Deficiency Anemia: Expert Review, Clinical Gastroenterology and Hepatology. (2024) 22, no. 8, 1575–1583, 10.1016/j.cgh.2024.03.046. 38864796

[104] Ning S. and Zeller M. P. , Management of Iron Deficiency, Hematology. American Society of Hematology. Education Program. (2019) 2019, no. 1, 315–322, 10.1182/hematology.2019000034. 31808874 PMC6913441

[105] Orhan M. F. and Büyükavci M. , Intravenous Iron Therapy for Children With Iron Deficiency Anemia, Journal of Pediatric Hematology. (2023) 45, no. 1, e56–E59, 10.1097/mph.0000000000002550. 36161971

[106] Crary S. E. , Hall K. , and Buchanan G. R. , Intravenous Iron Sucrose for Children With Iron Deficiency Failing to Respond to Oral Iron Therapy, Pediatric Blood and Cancer. (2011) 56, no. 4, 615–619, 10.1002/pbc.22930, 2-s2.0-79551640526. 21298748 PMC3079959

[107] Surico G. , Muggeo P. , Muggeo V. et al., Parenteral Iron Supplementation for the Treatment of Iron Deficiency Anemia in Children, Annals of Hematology. (2002) 81, no. 3, 154–157, 10.1007/s00277-001-0418-3, 2-s2.0-0036944658. 11904741

[108] Ahsan Baig M. M. , Batool S. , Aslam T. et al., Efficacy and Safety of Intravenous Iron in Children With Iron Deficiency Anaemia Poorly Compliant to Oral Iron Therapy, Journal of Ayub Medical College, Abbottabad. (2022) 34, no. 2, 317–320, 10.55519/jamc-02-9365. 35576294

